# Experimental evaluation of cobalt adsorption capacity of walnut shell by organic acid activation

**DOI:** 10.1038/s41598-023-33902-9

**Published:** 2023-05-05

**Authors:** Adnan Irshad, Muhammad Atif, Ambreen Ghani, Basharat Ali, Sheikh Asrar Ahmad, Musinguzi Alex

**Affiliations:** 1 Chemistry Department, University of Education Lahore (Vehari Campus), Vehari, Punjab Pakistan; 2 Busitema University, Tororo, Uganda

**Keywords:** Materials science, Environmental chemistry, Green chemistry, Materials chemistry

## Abstract

Cobalt, from industrial waste and nuclear laundry, possess health risk to human beings, animals and plants. Number of methods, other than adsorption, have been reported in literature for Co removal from waste water. In this research walnut shell powder after modification has been utilized for Co adsorption. First step of modification involved chemical treatment by four different organic acids for 72 h. Samples were collected at 24, 48 and 72 h. Second step involved thermal treatment of 72 h samples. Unmodified and modified particles have been analyzed by chemical methods and instruments i.e. UV spectrometer, FTIR, cyclic voltammetry (CV) and microscopic imaging. Thermally treated samples have shown augmented Co adsorption. CV analysis showed thermally treated samples with better capacitance. Particles modified by oxalic acid presented better Co adsorption. Oxalic acid treated particles activated for 72 h with thermal treatment provided maximum adsorption capacity 1327 ± 20.6 mg/g against Co(II) at pH 7, stirring 200 rpm, initial concentration 20 ml, adsorbent dosage (5 mg) and contact time 240 min at room temperature.

## Introduction

One of the primary concerns that mankind confronting today is water pollution. Heavy metals have been significantly contributing to water contamination. Due to their extreme bioaccumulation, a number of heavy metals, including Pb, U, As, Zn, Cu, Hg, Cd, Ni, Co, etc., are having a substantial negative influence on mankind^1,2^ but Co is a severe water contaminant because its excessive exposure affects not only the human beings but also plants and animals^3,4^. In 2011, more than 109,000 metric tons of Co were produced, while more than 75,000 metric tons were consumed^5^. Mineral ores in the form of sulphide and arsenide are the main source of cobalt production^6^. These ores are then further processed for use in various industries. Cobalt is widely used as cathodic materials in batteries, super alloys in jet engines, carbide materials in wear-resistant and cutting tools, magnetic alloys, feed additives, pigments, glass decolorizers, among other applications^5,7,8^. Such widespread use opens up a wide channel for pollutant sources. In addition to industrial waste, cobalt dumping has a significant impact on water contamination, endangering not just the civic sector but also the agricultural and livestock industries.

Massive amounts of cobalt can have harmful consequences on human health, including aberrant thyroid function, excessive red blood cell formation, contact dermatitis, and cardiovascular damage. Asthma, pneumonia, and wheezing are among the effects it has on the lungs^9–11^ while increased Co(II) ion concentration in plants results in chlorosis and necrosis, limits the development of roots, and reduces their ability to absorb nutrients and water^3^. Animals may have cardiac collapse, neurological problems, and reproductive problems if they absorb more Co(II) ions than is safe^12^.

Removal of such heavy metals as cobalt is crucial because they pose dangers to the ecosystem and might cause severe environmental harm. Many conventional methods have been used to remove cobalt, including chemical reduction^13^, chemical precipitation^14^, coagulation^15^, the ion exchange process^16^, electrodeposition^17^, electrocoagulation^18^, nano-filtration^19^, microfiltration^20^, ultrafiltration^21^, distillation^22^, forward osmosis^23^, electro-dialysis^24^, and liquid membranes^25^. However, these methods are expensive and produce sludge, so in this research adsorption method has been utilized for its simplest, least expensive, and least labor-intensive^26^ a credible and affordable process that does not create this waste.

Adsorption on carbonaceous materials is well known approach^27,28^. Adsorption of heavy metals on biowaste derived carbonaceous matter is thought to be more cost-effective than commercial activated carbon, most probably owing to the conductive nature^29^. As a byproduct of widespread industrialization, large quantities of carbonaceous wastes are produced from the processing of many carbon-based materials. One such example is agricultural waste, which arises from the processing of food sources like seeds used in oil extraction, maize used as a tanning agent, and countless other applications of vegetables and fruits in the production of a vast array of consumer goods^30–32^. Such biowastes are particularly useful in a variety of applications, including adsorption, thanks to their structural characteristics and synthetic approaches^33^. Variety of aquatic pollutants including Pb^34^, As^35^, Hg^36^, rhodamine B^37^, methylene orange^38^, malachite green^39^, etc., could be removed through adsorption using facile and complex adsorbents^40,41^. Because of their high surface area and ease of functionalization, biowastes are incorporated into the production of a wide range of materials such as catalysts^42^, double layer capacitors^43^, polymers^44^ nanoparticles^45^, supercapacitors^46^, etc. Different biowastes have been modified chemically or thermally to remove heavy metals including rice husk^47^, banana peel^48^, papaya peel^49^, kernel shell^50^, prawn shell^51^, bamboo bark^52^ and many more. Agricultural biowaste such as apricot stone^53^, hazelnut shell^54^, buckwheat hulls^55^, lemon peel^56^, orange peel^57^, potato peel^58^, rice husk^59^, have been chemically or thermally treated to improve surface chemistry and textural characteristics that are ideal for co-adsorption. Because of its exceptional textural characteristics, walnut shell has been reported for high surface catalyst and nanoparticles^60^. This work presents a simple, novel, and eco-friendly method for producing metal adsorbents from walnut shell by treating it with four different organic acids and/or heat. The resulting adsorbents for Co(II) removal are effective, inexpensive, and benign to the environment.

## Materials and methods

### Materials

Acetic acid (sigma-aldrich; 99.7%), benzoic acid (sigma-aldrich; 99.5%), oxalic acid dihydrate (sigma-aldrich; 99.5%), salicylic acid (merck & Co; 99.5%), 2-propanol (sigma-aldrich; 99.5%), acetone (sigma-aldrich; 99.5%), ammonium thiocyanate (sigma-aldrich; 99.5%), cobalt chloride (sigma-aldrich; 98%) have been utilized, as received.

### Sample preparation

**Scheme 1:**
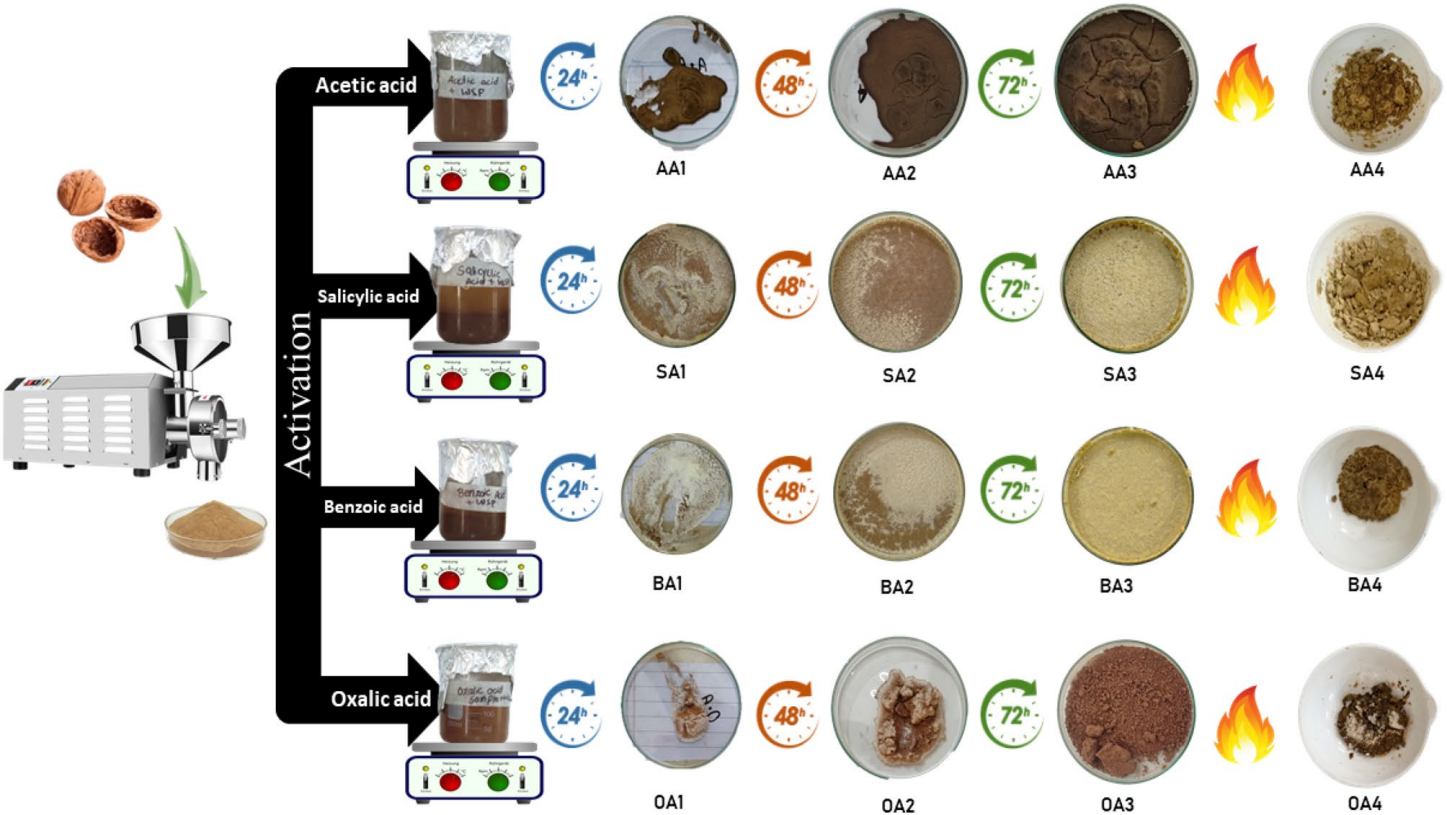
Methodology scheme.

Walnut shell, after cleaning, has been crushed into powder (WP), sample preparation has been done as per data given in Table 1 (Scheme 1).

**Table 1:** Sample preparation strategy and nomenclature.

Sr. No	Sample ID	Composition				Activation time (hrs)	Carbonization temp (℃)
		Particle	Modifier	Particle:Modifier (wt:wt)	Solvent (120 mL)		
1	WP	WP	Nil	Nil	Nil	Nil	rt
2	AA1	WP	Acetic Acid	1:1	Water	24	rt
3	AA2	WP	Acetic Acid	1:1	Water	48	rt
4	AA3	WP	Acetic Acid	1:1	Water	72	rt
5	AA4	WP	Acetic Acid	1:1	Water	72	550
6	BA1	WP	Benzoic Acid	1:1	2-propanol	24	rt
7	BA2	WP	Benzoic Acid	1:1	2-propanol	48	rt
8	BA3	WP	Benzoic Acid	1:1	2-propanol	72	rt
9	BA4	WP	Benzoic Acid	1:1	2-propanol	72	550
10	OA1	WP	Oxalic Acid	1:1	Water	24	rt
11	OA2	WP	Oxalic Acid	1:1	Water	48	rt
12	OA3	WP	Oxalic Acid	1:1	Water	72	rt
13	OA4	WP	Oxalic Acid	1:1	Water	72	550
14	SA1	WP	Salicylic Acid	1:1	2-propanol	24	rt
15	SA2	WP	Salicylic Acid	1:1	2-propanol	48	rt
16	SA3	WP	Salicylic Acid	1:1	2-propanol	72	rt
17	SA4	WP	Salicylic Acid	1:1	2-propanol	72	550

WP has been treated with each organic acid solution under constant stirring. Samples have been collected after 24, 48 and 72 h and oven dried at 60 °C for 12 h. Samples collected after 72 h have been divided into 2 parts from which one has been carbonized at 550 °C for 3 h in muffle furnace at ramp rate of 8.67 °C/min under oxidizing environment. All prepared samples have been saved in glass vials for further analysis.

## Characterization

Prepared samples have been characterized by below-mentioned techniques

FTIR spectra of prepared samples have been recorded using FTIR-ATR (IR-Spirit Shimadzu, Japan) within the range of 4000–500 cm^−1^.

Micrographic pictures has been taken using “IRMECO GmbH & CO. Model No. IM-910” on calibration slide (0.01–0.1 mm) with 8X resolution to evaluate average size of agglomerates.pH is the negative log of concentration of hydrogen ions. pH is an important factor which has the ability to alter the obtained results of a reaction therefore it is important to keep tracking pH of prepared samples. Prepared samples (0.005 g each) in 10 ml of distilled water stirred at 450 rpm for 1 h, have been subjected to pH tester “Milwaukee pro waterproof pH/temp”, and conductivity by using “JENWAY 4510 conductivity meter”.

Dispersion stability of prepared samples (0.005 g) has been tested in distilled water (10 mL). After 1 h constant stirring, time has been noted for samples to completely settle down^61^. CV of WP and prepared samples have been performed using “Gamry Reference 3000” with potential window (− 0.6 to + 0.6) under scan rate of 10 mV/sec in 1 M KOH.

### Adsorption analysis

Adsorption analysis has been done as per method reported in literature^62^, with a slight modification of using 10 ml of 3000 ppm Co solution and 5 mg adsorbent, sampling at 15 min’ interval, and analyzed by using UV–vis spectrophotometer (CECIL, CE74000) at λ_max_ 625 nm. Percentage removal (Eq. 1) and adsorption capacity (Eq. 2) have been calculated by the following equations.

1 $$\text{Percentage Removal}(\text{\%R})=\left(\frac{{\text{C}}_{\text{i}}-{\text{C}}_{\text{t}}}{{\text{C}}_{\text{i}}}\right)\times 100$$

2 $${\text{Adsorption}}\;{\text{Capacity}}\;({\text{q}}_{{\text{t}}} ) = \left( {{\text{C}}_{{\text{i}}} - {\text{C}}_{{\text{t}}} } \right) \times {\raise0.7ex\hbox{${\text{V}}$} \!\mathord{\left/ {\vphantom {{\text{V}} {\text{M}}}}\right.\kern-0pt} \!\lower0.7ex\hbox{${\text{M}}$}}$$

Here, C_i_ and C_t_ are the initial and final concentrations of adsorbate whereas M is mass of adsorbent in grams and V is volume of metal solution in liters used for adsorption analysis. Co(II) concentration (mg/L) calculations was done by Eqs. (3) and (4), adopted from a reported method^62^.

3 $$c=A\times 0.0477$$

4 $$c=\frac{10}{n}\times \frac{A}{0.0477}$$

whereas “c” is concentration in mg/L, A is absorbance, “n” is volume (ml) taken for analysis.

## Results and discussion

### FTIR analysis

**Figure 1:**
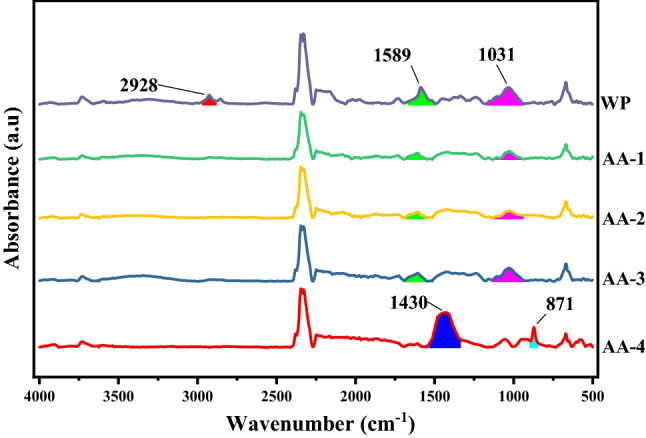
FTIR absorption spectra of AA activated samples. OriginPro 2022 (64-bit) SR1 v9.9.0.225 https://www.originlab.com/index.aspx?go=Support&pid=4440.

**Figure 2:**
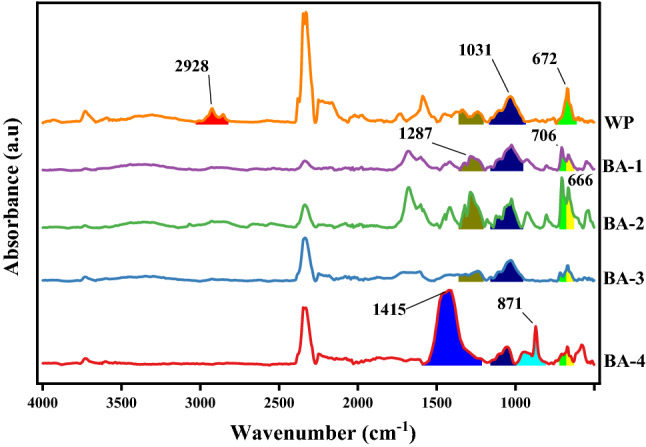
FTIR absorption spectra of BA activated samples. OriginPro 2022 (64-bit) SR1 v9.9.0.225 https://www.originlab.com/index.aspx?go=Support&pid=4440.

**Figure 3:**
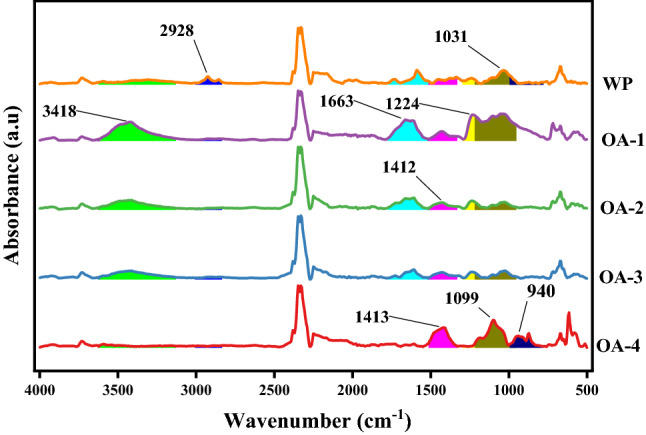
FTIR absorption spectra of OA activated samples. OriginPro 2022 (64-bit) SR1 v9.9.0.225 https://www.originlab.com/index.aspx?go=Support&pid=4440.

**Figure 4:**
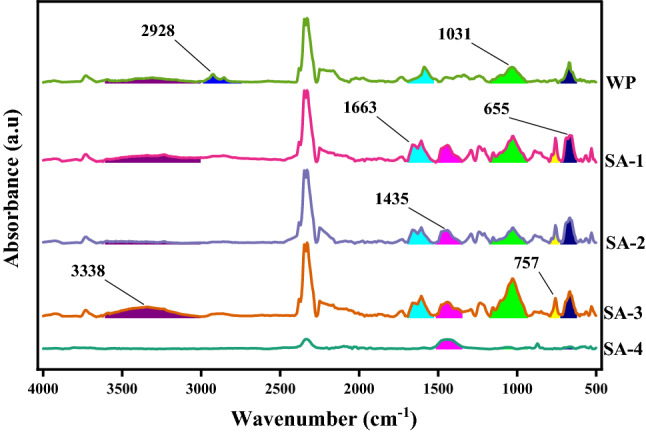
FTIR absorption spectra of SA activated samples. OriginPro 2022 (64-bit) SR1 v9.9.0.225 https://www.originlab.com/index.aspx?go=Support&pid=4440.

FTIR absorbance spectra (Figs. 1, 2, 3 and 4) of WP before and after activation with organic acids have been recorded within the range of 4000–500 cm^−1^.

#### Acetic acid (AA) activated samples

FTIR absorbance spectra (Fig. 1) of modified samples presented declining peaks at 2928 cm^−1^ (sp^3^ C–H str), 1589 cm^−1^ (C=O sr) and 1031 cm^−1^ (alcoholic C–O str), signposting partial utilization of these functionalities at room temperature (physical adsorption of AA on WP) and complete utilization at high temperature i.e. sample AA4 (augmented reaction kinetics). Reduction of 1031 cm^−1^ peak suggests condensation, resulting in ester formation (peak at 871 cm^−1^), counter confirmed by the appearance of new peak at 1430 cm^−1^ representing C–H asymmetric bending of AA methyl group.

#### Benzoic acid (BA) activated samples

In FTIR spectra (Fig. 2) of modified samples peak at 2928 cm^−1^ (sp^3^ C–H str) disappeared and new peaks at 1680 cm^−1^ (C=O str), 1412 cm^−1^ (C–C str in aromatic rings), 1287 cm^−1^ (C–O str of ester) and 706 cm^−1^ (benzene derivatives) appeared confirming benzene induction on WP surface. Gradual reduction of 1031 cm^−1^ (alcoholic C–O str) and increase of 1287 cm^−1^ and 871 cm^−1^ (ester) confirmed condensation. Covalent connection of BA and WP got confirmed through peaks at 706 cm^−1^ (benzene derivatives) and 1412 cm^−1^ (C–C str in aromatic rings), along with splitting of 672 cm^−1^ peak into two peaks 706 & 666 cm^−1^ (benzene derivatives). Thermal treatment increased peak area at 1415 cm^−1^ (C–H bending) and peak height at 871 cm^−1^ (ester) evidencing condensation.

#### Oxalic acid (OA) activated samples

3418 cm^−1^ (O–H str) peak expansion on chemical treatment and disappearance on heat treatment established OA physical and chemical adsorption on WP, respectively (Fig. 3). After activation peak at 2928 (sp^3^ C–H str) disappeared presenting utilization of functionality, whereas appearance of three prominent peaks at 1663 cm^−1^ (Carbonyl C=O str), 1099 cm^−1^ and 1413 cm^−1^ (Ester C–C str) confirmed the formation of new oxygen containing functionalities due to OA induction on WP surface. Thermal oxidation shifted peak at 940 cm^−1^ (carboxylic O–H bend) and developed new peak at 1412 cm^−1^ (C–C str).

#### Salicylic acid (SA) activated samples

FTIR spectra (Fig. 4) meant 2928 cm^−1^ peak (sp^3^ C–H str) as reaction site as for its disappearance after modification. Chemical modification budded peaks at 3338 cm^−1^ (O–H str), 1663 cm^−1^ (carbonyl C–O str) and 1435 cm^−1^ (aromatic C–C str), 1031 cm^−1^ (Ester C–O str), 757 cm^−1^ (aromatic C–H bending) and 655 cm^−1^ (Benzene derivative), which on thermal treatment got completely disappeared indicating thermal disintegration.

### Magnetic behavior

**Figure 5:**
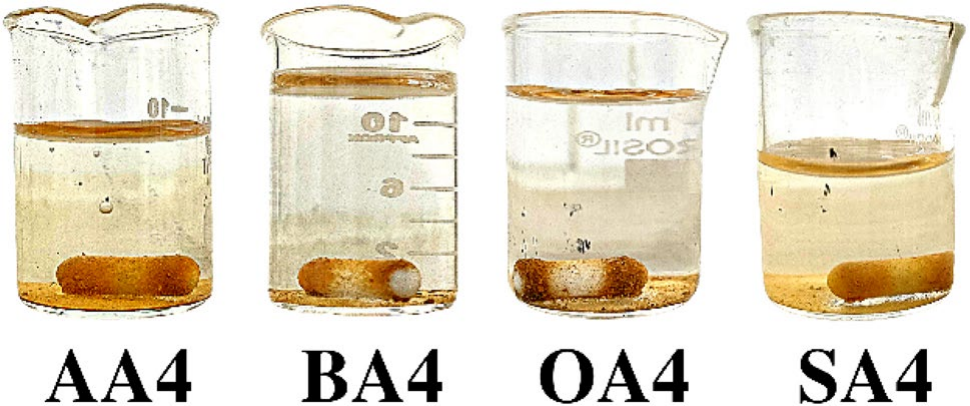
Magnetic behavior of thermally treated samples.

Thermal treatment has induced magnetic behavior in samples (Fig. 5) which otherwise is absent in chemically activated samples. It could be evaluated that thermal treatment at very slow ramp rate have degraded oxidizable contents on the surface of samples which led to mineral exposure from within material which enhanced magnetic pull.

### Average agglomerate size

Comparison of unmodified and modified samples have shown a change in agglomerate sizes (Table 2). It has been observed that increased activation time augmented agglomerate size as well as conductivity, which may be attributed to increased functionalities and subsequently increased attractive forces upon samples’ surface. However, thermal treatment has resulted in reduced agglomerate size, which may be attributed to the fact that oxidizable organic contents decomposed resulting in reduced attractive forces. The same phenomena have been observed in OA activated samples. Agglomeration trend in BA and SA modified samples has been observed different than OA and AA activated samples, due to induced aromatic ring on WP surface. Maximum average agglomerate size has been obtained from OA3 whereas SA4 showed least agglomeration.

**Table 2:** Physico-chemical, acidic contents and agglomerate size of prepared samples.

Sr. #	Sample ID	Physico-chemical		Agglomerate size (mm)
		Conductivity (µS)	pH	
1	AA-1	0.55	7.1	0.015
2	AA-2	0.55	7.1	0.020
3	AA-3	0.58	7.6	0.034
4	AA-4	0.54	7.2	0.030
5	BA-1	0.50	3.8	0.020
6	BA-2	0.50	3.9	0.024
7	BA-3	0.53	4.1	0.027
8	BA-4	0.52	7.3	0.020
9	OA-1	0.66	2.7	0.030
10	OA-2	0.76	2.8	0.040
11	OA-3	0.79	2.9	0.045
12	OA-4	0.55	7.1	0.025
13	SA-1	0.69	3.5	0.025
14	SA-2	0.80	3.6	0.035
15	SA-3	0.84	4.4	0.040
16	SA-4	0.52	7.2	0.007
17	WP	0.48	6.4	0.015

### Physico-chemical analysis

pH and conductivity results are given in Table 2. OA-1 has shown the lowest pH (2.7) and BA-4 the highest (7.3). It has been observed that activation time as well as thermal treatment augmented pH value. Conductivity trend have been found similar among prepared samples, but always higher than WP.

### Dispersion test

**Figure 6:**
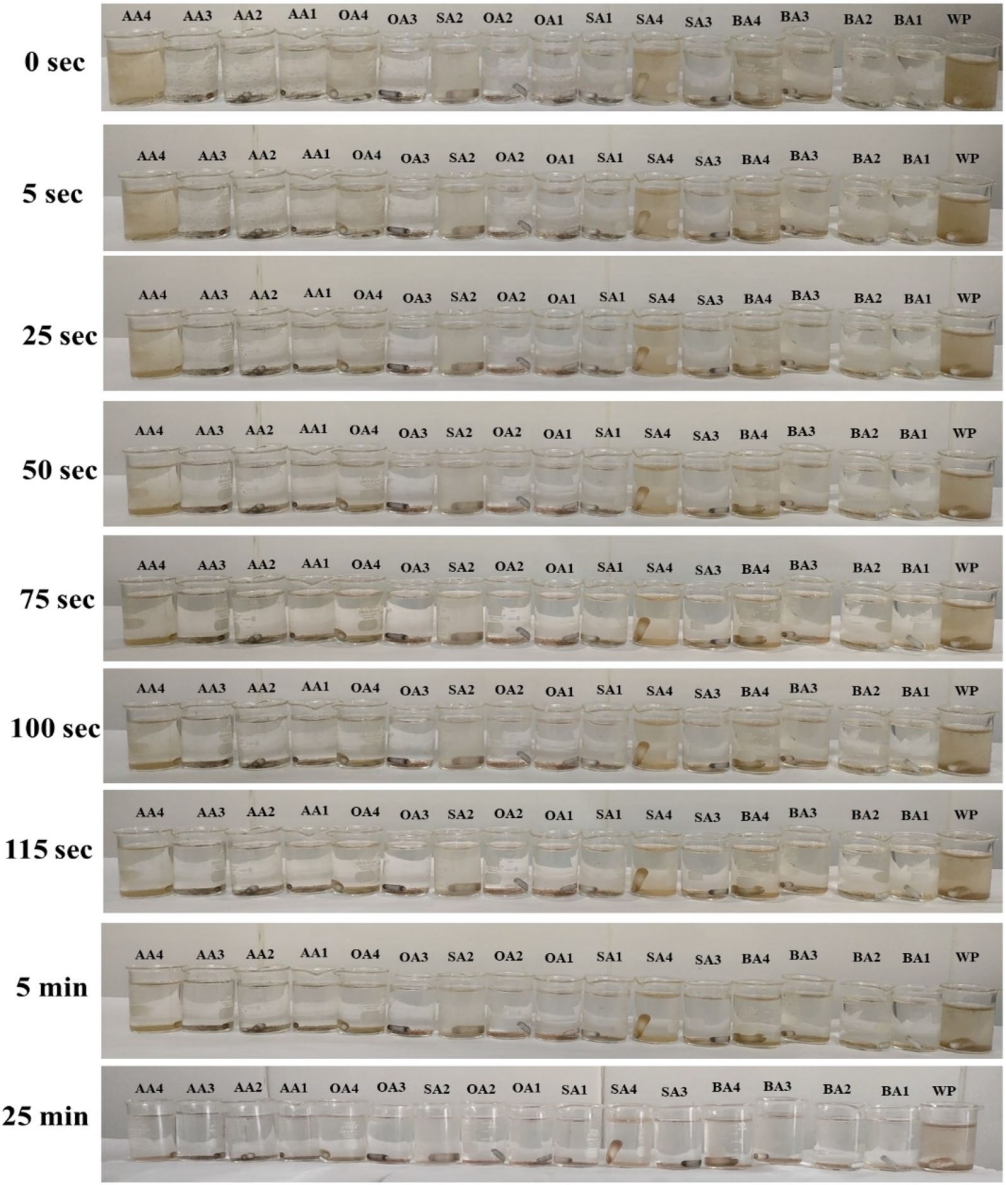
Dispersion of modified and unmodified samples at different time intervals.

Dispersion analysis of unmodified and modified samples (Fig. 6) showed a quick settling of prepared samples (approx. 2 min) in comparison to untreated material (25 min). Fast settling of modified material showed hydrophobicity induced with surface chemistry change (see pH and FTIR), as well as increased agglomerate size due to condensation of surface functionalities incorporated on particle surfaces after modification.

### Cyclic voltammetry

**Figure 7:**
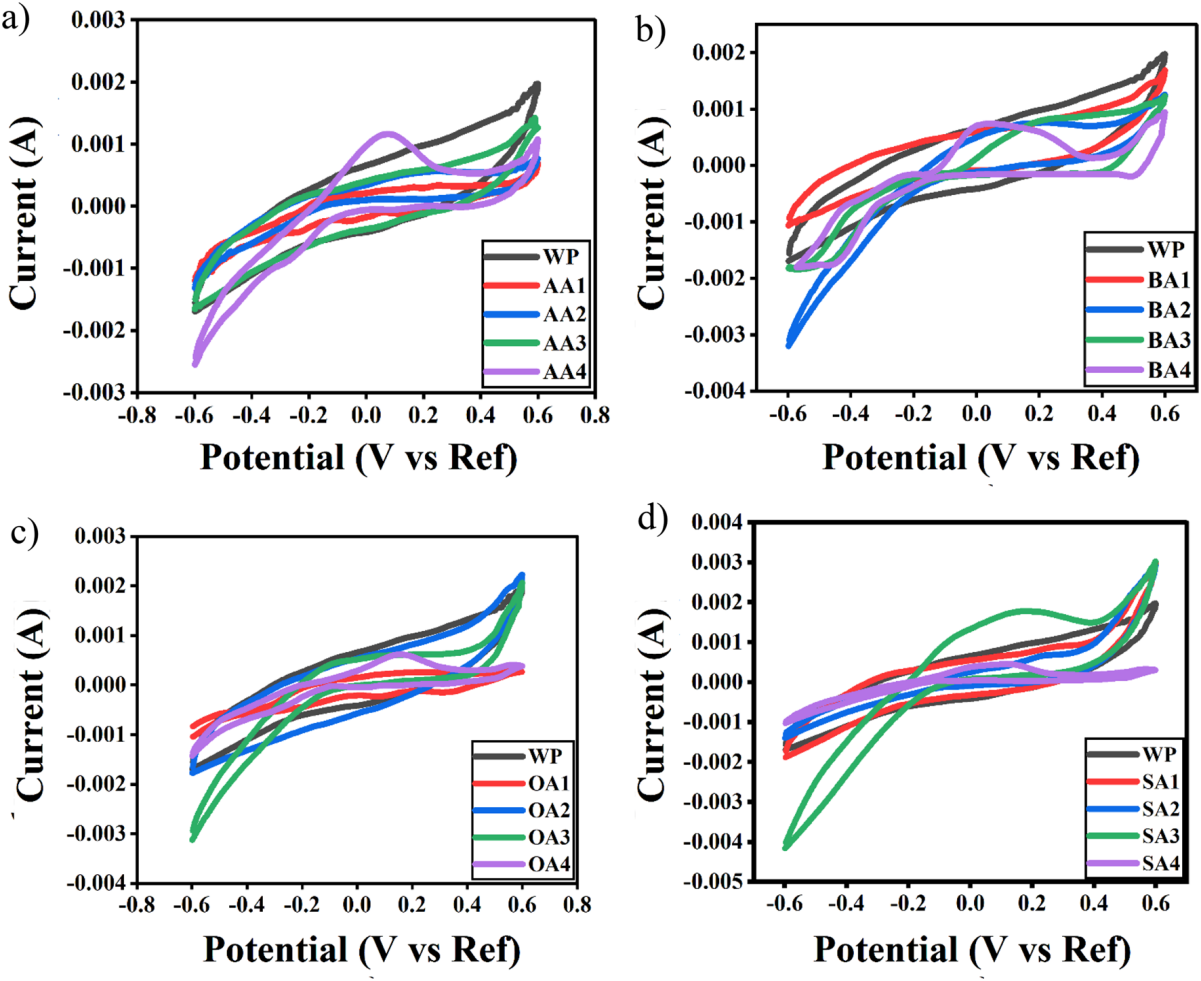
CV curves (Current vs Potential) of prepared adsorbents activated with (**a**) AA (**b**) BA (**c**) OA (**d**) SA in voltage range from − 0.6 to + 0.6 at scan rate 10 mV/s in 1 M KOH. OriginPro 2022 (64-bit) SR1 v9.9.0.225 https://www.originlab.com/index.aspx?go=Support&pid=4440.

Cyclic voltammetry (CV) has been done to explain possible adsorption mechanism of modified samples (Fig. 7a–d). It has been observed that thermal treatment has intensified reduction peak allowing material to create stable interaction with metal cations. As per Fig. 7a, AA4 has highest surface conductivity evident from the highest peak, which marks it applicable for metal cations interaction. On the contrary, AA2 has shown the least conductance making its surface less interactive with metal cations. BA activated samples (Fig. 7b) have also shown similar pattern i.e. thermal treatment augmented conductive response than chemically modified samples. In OA activated samples (Fig. 7c) consistency in charge carrying capacity and activation time has been observed, yet still thermal treatment has developed reduction peak indicating material suitability for reducing metals. In SA activated samples (Fig. 7d), SA3 showed highest reduction peak which may be attributed to additional surface functionalities, as presented in FTIR (Fig. 4).

**Figure 8:**
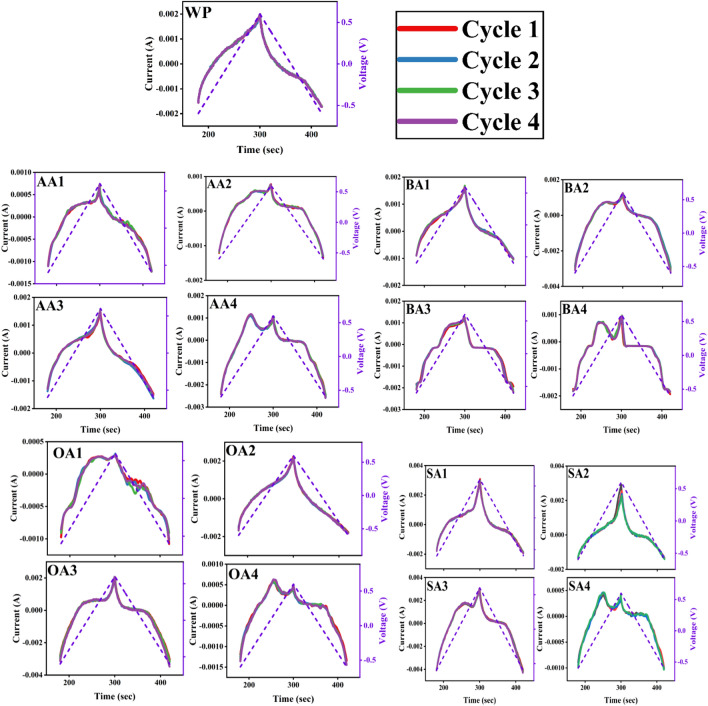
Charge–discharge analysis (Current vs Time) of unmodified and modified samples. OriginPro 2022 (64-bit) SR1 v9.9.0.225 https://www.originlab.com/index.aspx?go=Support&pid=4440.

Charge discharge behavior for unmodified and modified samples have been analyzed, through comparison of cyclic voltammograms taken at different time intervals (Fig. 8), and no change in current carrying capacity of unmodified and modified samples has been observed, verifying reusability of material.

### Adsorption analysis

Adsorption capacity of prepared samples against Co^2+^ has been tested. Prepared samples have been dipped in metal solution for a pre-determined time with constant stirring. Metal solutions have been tested using UV–Vis spectrophotometer after specific time interval at wavelength of 625 nm.

**Figure 9:**
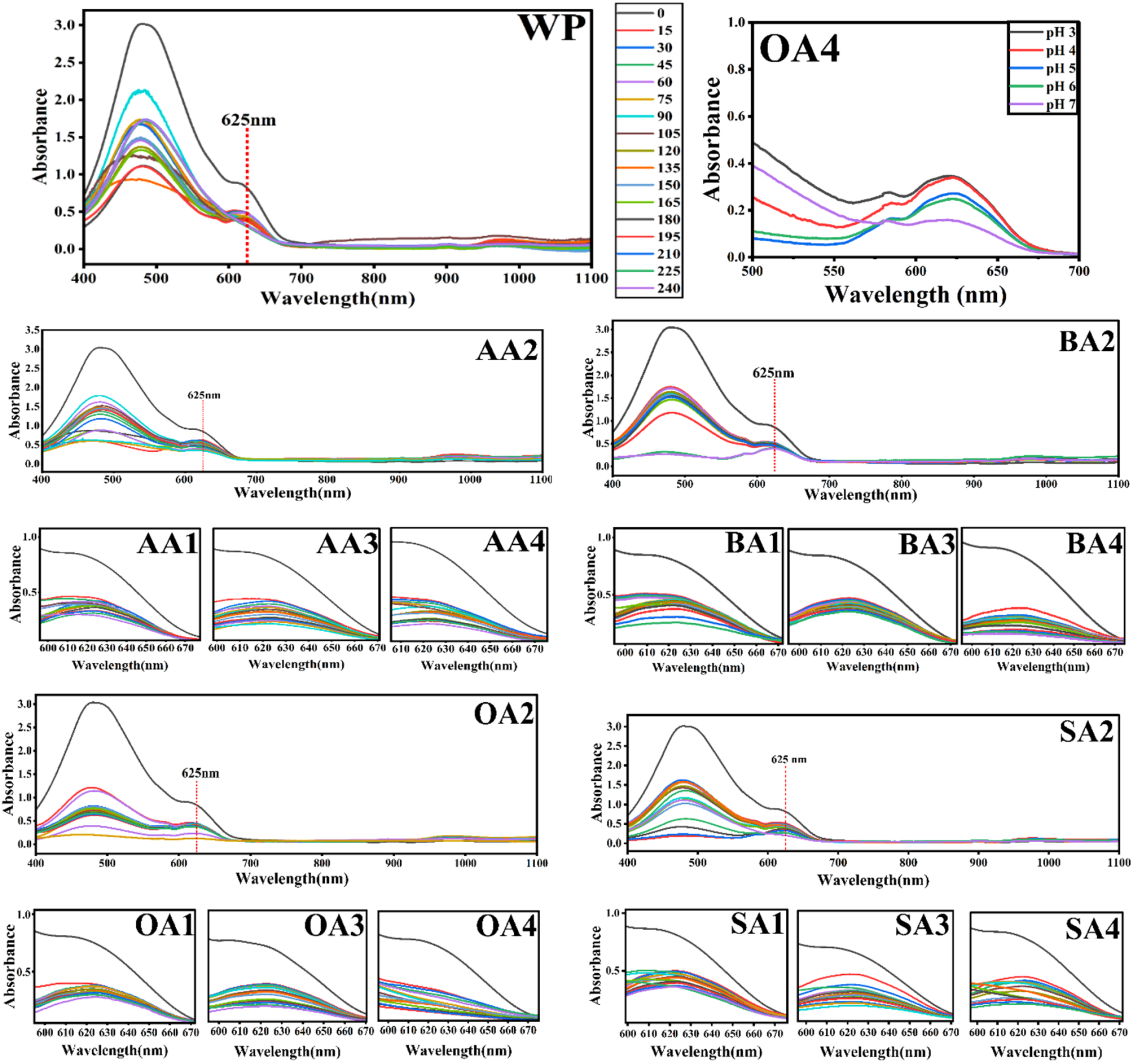
UV–vis spectra of cobalt solution after treating with unmodified and modified samples. OriginPro 2022 (64-bit) SR1 v9.9.0.225 https://www.originlab.com/index.aspx?go=Support&pid=4440.

UV analysis of prepared cobalt solution and its calculations have been performed by following the method already reported in literature^62^. UV–vis experimentation has been carried out at scan speed 1 nm/sec and scale at 25 nm/cm. All the samples have been analyzed between 400 and 1100 nm wavelength range (Fig. 9). Data showed a reciprocal relation between contact time and absorbance, indicating Co-adsorption. Furthermore, OA4 has been tested at different pH from 3 to 7 (Fig. 17) and found to have incremental effect on adsorption with high pH. Experiment designed in such a way that values have been obtained from each data set with replication number of 3 whereas accuracy determined to be the closest to expected results.

#### FTIR after adsorption

FTIR spectra of unmodified and modified samples have been taken after adsorption and found to have vibrations in 700–400 cm^−1^ range due to metal—oxygen bond^63^.

**Figure 10:**
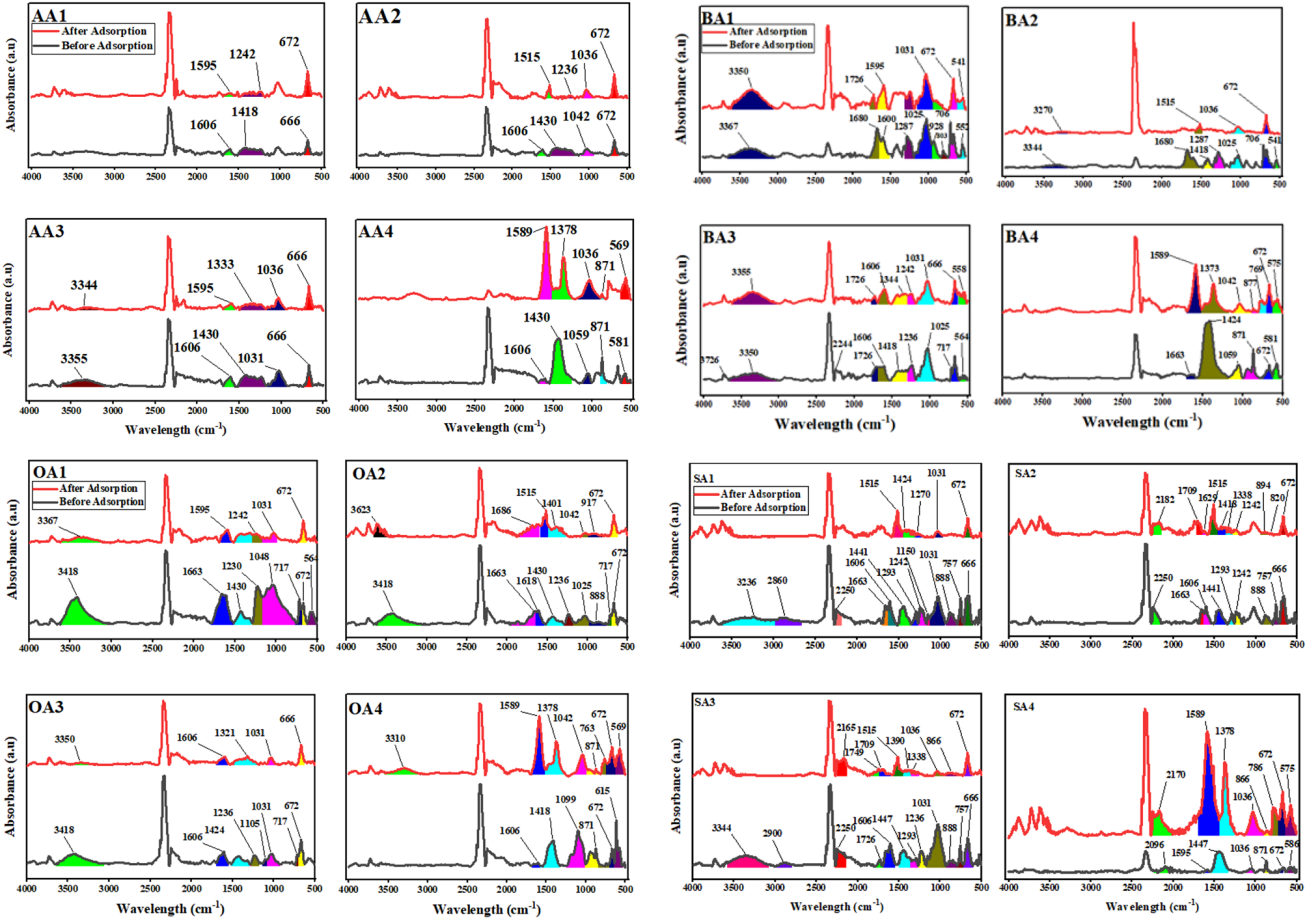
FTIR of modified and unmodified samples before and after Co(II) adsorption. OriginPro 2022 (64-bit) SR1 v9.9.0.225 https://www.originlab.com/index.aspx?go=Support&pid=4440.

AA activated samples have shown significant interaction against Co(II) ions (Fig. 10). Disappearance of peak at 1430 cm^−1^ (COO^−^ str) in all variants of AA activated samples indicated Co(II) ions interaction with carbonyl functionalities present on sample surface which could be counter confirmed through enhanced intensity of peaks at 672 cm^−1^ and 666 cm^−1^, attributed to metal-O bond formation^63,64^. All AA activated samples, after adsorption, have shown peak shifting form 1606 cm^−1^ (Carboxylic; COO^−^ str) to 1505–1595 cm^−1^, confirming metal chelation with carbonyl functionalities^65,66^. From the data it could be evaluated that proton shifting and ion exchange process helped in adsorption of cobalt ions on AA samples’ surface.

BA samples (Fig. 10) showed weak interaction with metal, most probably due to their complex surface chemistry. Peak shifted from 1606 to 1515–1590 cm^−1^ (COO^−^ str), from 1025 to 1031–1036 cm^−1^ (formats; CO–O str) with decreased intensity^67^ and peak appearance at 672 cm^−1^ (metal-O) confirmed cobalt adsorption on carbonyl and hydroxyl groups^63^. Broadening of peak (OH str) designated to water molecules coordinated with cobalt ions, chelated with OH group of the benzoic acid^68^.

Chemically modified OA samples (Fig. 10) consumed hydroxyl groups for cobalt adsorption as confirmed by peak reduction at 3418 cm^−1^ (O–H str) as well as peak appearance below 700 cm^−1^ (metal-O vibrations). Besides, peak shifting from 1600–1700 cm^−1^ to 1500–1600 cm^−1^, peak disappearance at 717 cm^−1^ (formats; O–C=O str) and 850–900 cm^−1^confirmed cobalt chelation with carbonyl groups.

SA samples (Fig. 10) consumed peaks at 1600–1200 cm^−1^ except a peak shift on 1515 cm^−1^ confirming carbonyl cobalt chelation. SA1 and SA3 showed peak reduction at 3220–3350 cm^−1^ (Carboxylic associated O–H str) after cobalt adsorption indicating consumption of OH group of salicylic acid present on the ring. Thermally treated sample after cobalt adsorption gave high intensity peaks at 1589 (aromatic; asymmetric COO^−^ str) and 1378 (phenolic O–M str) indicating metal interaction with carbonyl and hydroxyl group.

**Figure 11:**
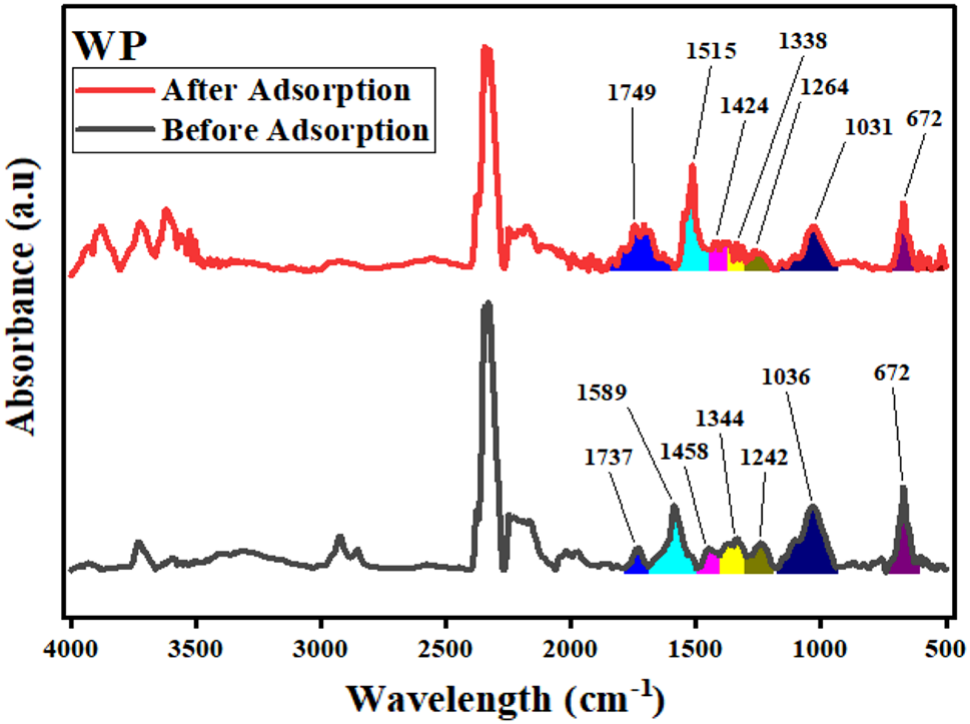
Comparative FTIR of WP before and after Co(II) adsorption. OriginPro 2022 (64-bit) SR1 v9.9.0.225 https://www.originlab.com/index.aspx?go=Support&pid=4440.

WP showed involvement of aromatic moieties along with hydroxyl and carbonyl groups in Co adsorption (Fig. 11). Peak shifted from 1589 to 1515 cm^−1^ gave promising evidence regarding cobalt chelation with aromatic compounds on WP surface. Peaks shifted from 1242 to 1264 cm^−1^ and from 1344 to 1338 cm^−1^ respectively counter verified involvement of carbonyl functionalities. Peak shifted from 1036 (formats; CO–O) to 1031 cm^−1^ along with altered peak at 672 cm^−1^ confirmed cobalt and oxygen bonding.

**Figure 12:**
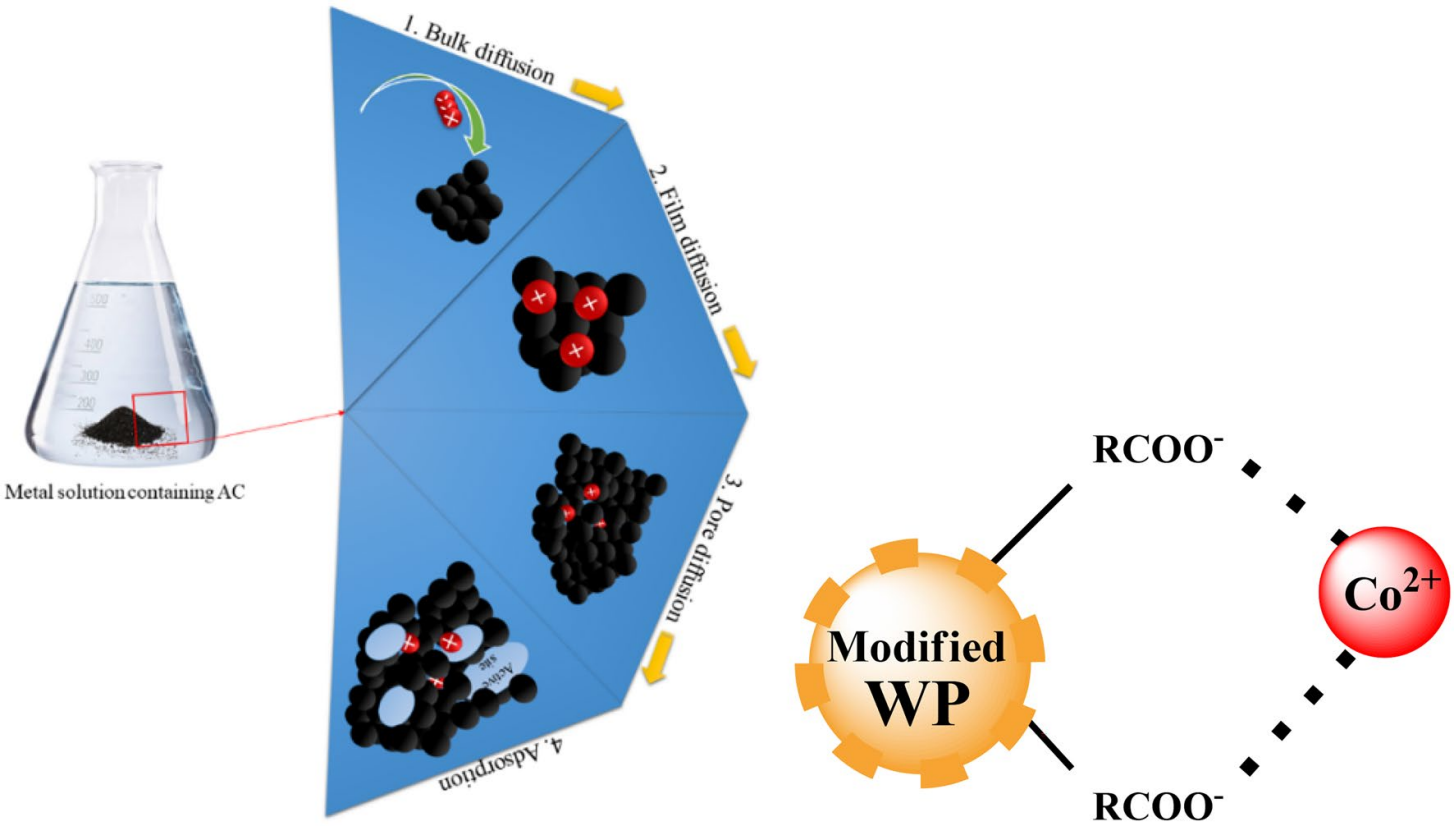
Surface interaction of Co(II) with prepared samples and its adsorption mechanism.

Surface interaction and adsorption mechanism of prepared samples against Co(II) metal through ion exchange process has been supposed to be as Fig. 12.

#### Adsorption capacities

**Figure 13:**
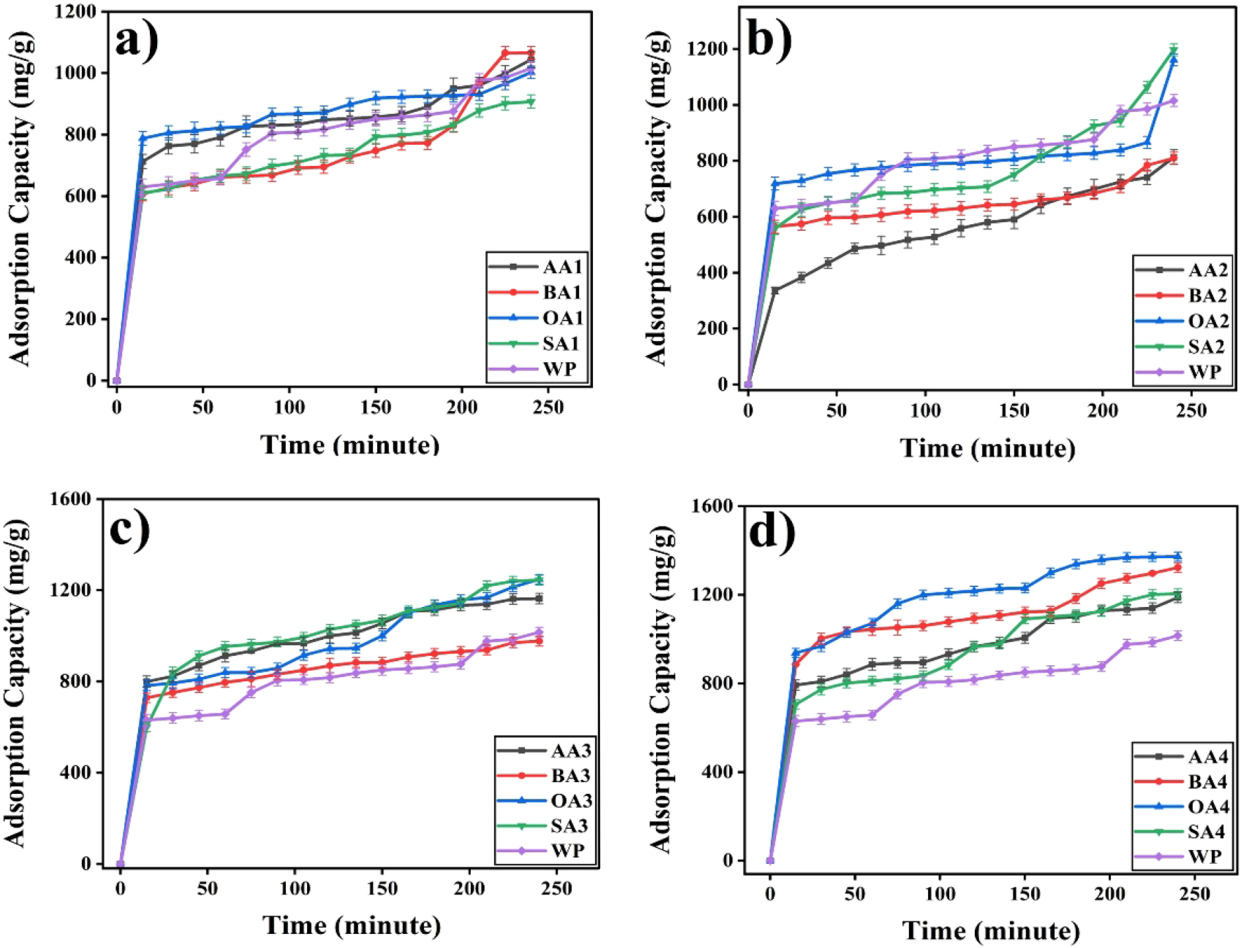
Effect of treatment time on adsorption capacity of (**a**) 24 h, (**b**) 48 h, (**c**) 72 h rt and (**d**) 72 h 550 degree C.

**Figure 14:**
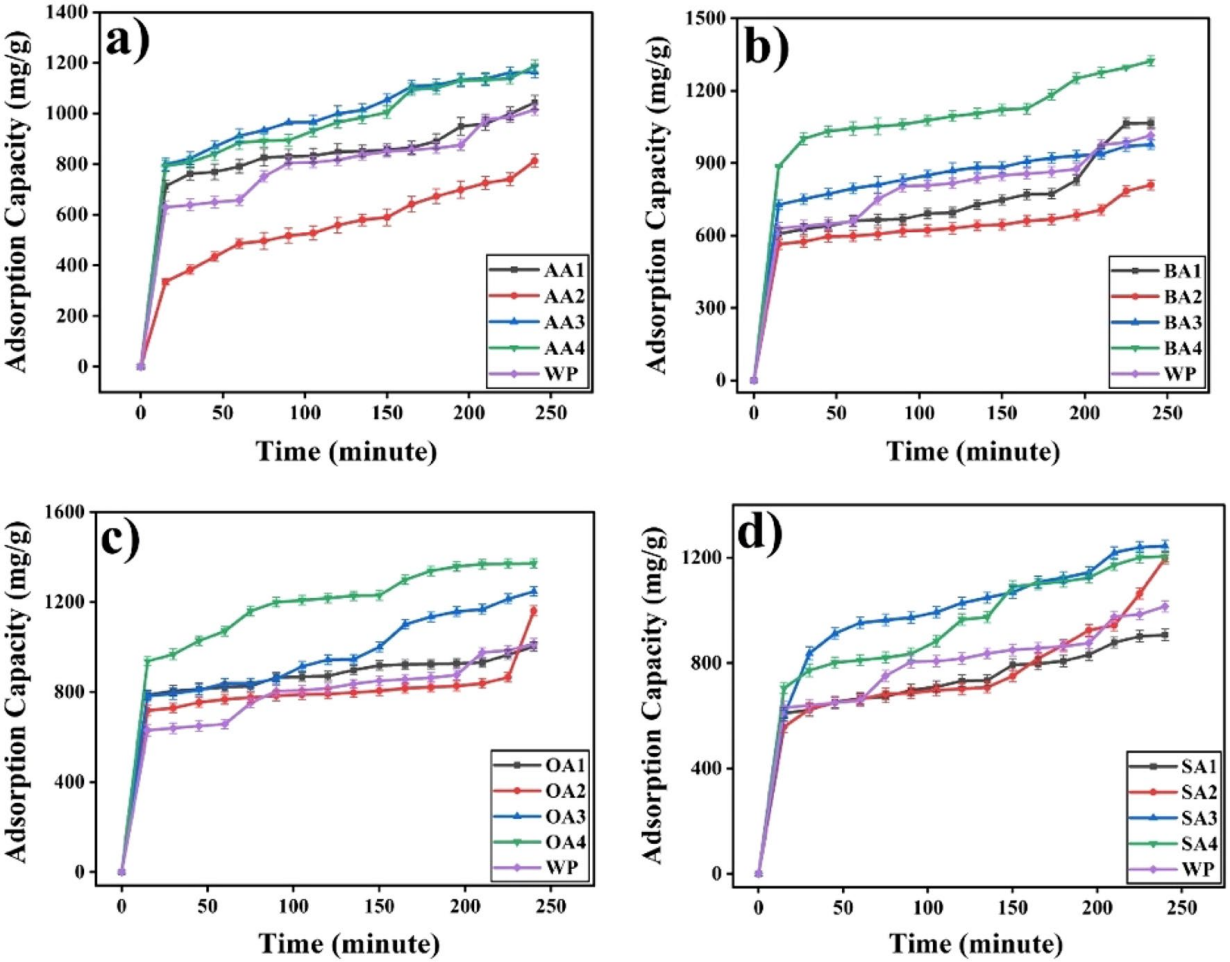
Effect of modifier on adsorption capacity (**a**) AA, (**b**) BA , (**c**) OA and (**d**) SA.

Prepared samples showed significant results against cobalt adsorption capacities (Table 3, Figs. 13 and 14) particularly in first 15 min of contact time, and later on adsorption process became slow due to less availability of active sites for Co adsorption. It has been observed that thermally treated samples except SA4 (Fig. 13d) showed maximum Co adsorption in comparison to other samples of same batch. OA4 showed maximum (1371.4 ± 20.6 mg/g) and BA2 (809.9 ± 21.1 mg/g) showed minimum adsorption capacity. Activation time played crucial role in adsorption behavior of samples. Samples with 24 h of activation (Fig. 14) have shown good adsorption against cobalt but 48 h activation have shown low adsorption which may be attributed to consumption of its surface functionalities i.e. dimerization of organic acids. Further activation (72 h) showed better adsorption among all chemically modified samples, whereas thermally treated samples showed the best adsorption, which may be attributed to thermal oxidation reactions on the surface of particles. Eventually, both surface chemistry and texture appear major. Optimum activation time has been found to be 72 h whereas thermal treatment enhanced samples’ surface area and oxygen content.

**Table 3:** Adsorption capacity and removal efficiencies of samples against heavy metal Co(II).

Sr.#	Sample ID	Adsorption Capacity(mg/g)	Removal %
1	WP	1015.5 ± 21.5	61.52 ± 1.3
2	AA1	1043.8 ± 26.5	63.23 ± 1.6
3	AA2	813.2 ± 25.9	50.25 ± 1.6
4	AA3	1163.2 ± 23.1	71.88 ± 1.4
5	AA4	1187.6 ± 23.6	71.95 ± 1.4
6	BA1	1065.4 ± 20.6	67.12 ± 1.3
7	BA2	809.9 ± 21.1	50.04 ± 1.3
8	BA3	976.9 ± 21.5	59.18 ± 1.3
9	BA4	1322.6 ± 21.5	80.13 ± 1.3
10	OA1	1002.2 ± 20.3	64.36 ± 1.3
11	OA2	1160.9 ± 21.5	70.33 ± 1.3
12	OA3	1247.5 ± 21.5	75.57 ± 1.3
13	OA4	1371.4 ± 20.6	86.40 ± 1.3
14	SA1	906.9 ± 21.5	54.95 ± 1.3
15	SA2	1197.2 ± 21.1	73.99 ± 1.3
16	SA3	1244.3 ± 21.5	75.39 ± 1.3
17	SA4	1204.9 ± 21.0	74.46 ± 1.3

Modifiers, effect surface chemistry and adsorption capacity of particles (Fig. 14). AA, BA, OA and SA despite of similar active functionality (-COOH) have differ in chemical structure.

#### Removal efficiencies

**Figure 15:**
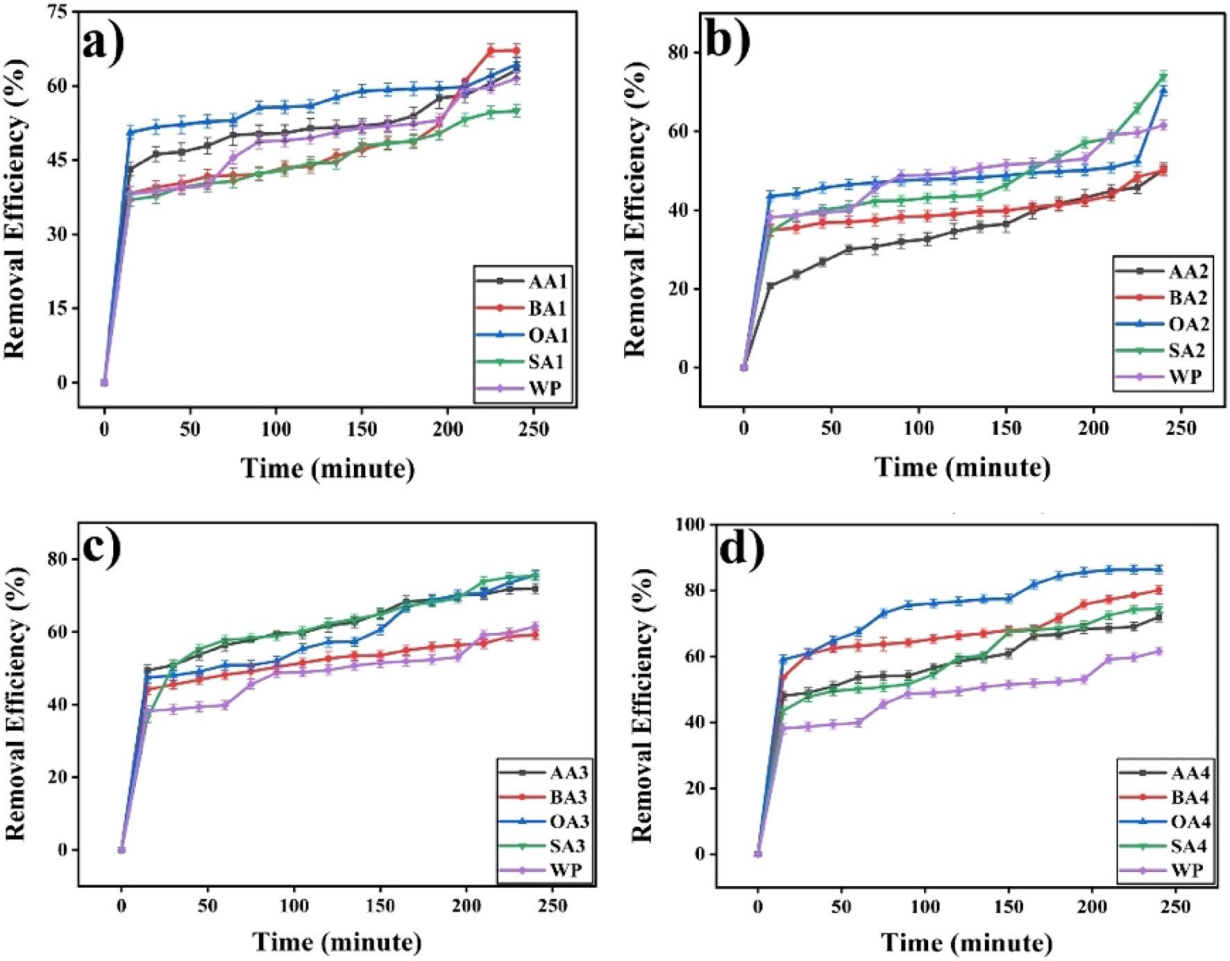
Removal Efficiencies of samples activated for (**a**) 24 h (**b**) 48 h (**c**) 72 h (**d**) Thermally treated against cobalt. OriginPro 2022 (64-bit) SR1 v9.9.0.225 https://www.originlab.com/index.aspx?go=Support&pid=4440.

**Figure 16:**
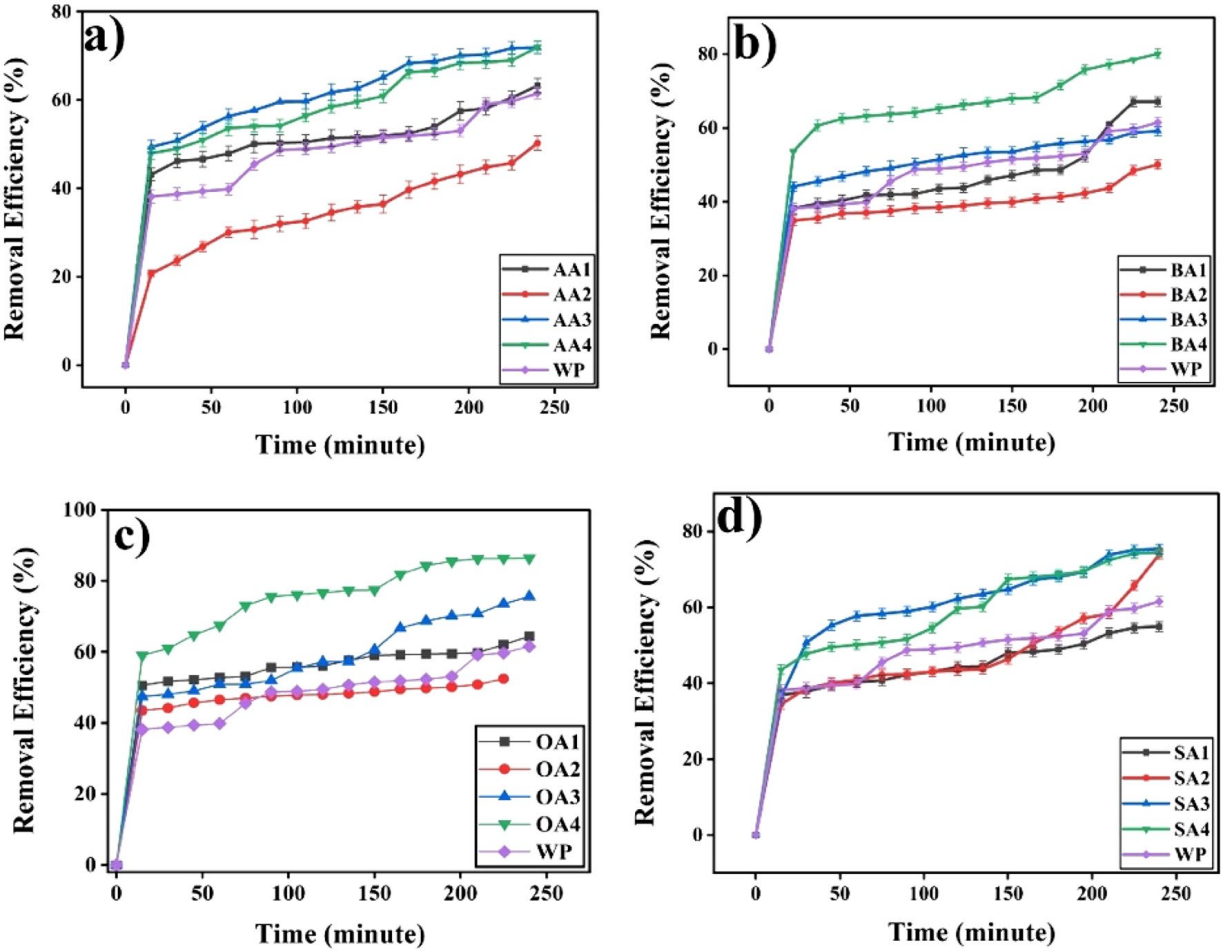
Removal efficiencies of samples activated with (**a**) AA (**b**) BA (**c**) OA (**d**) SA. OriginPro 2022 (64-bit) SR1 v9.9.0.225 https://www.originlab.com/index.aspx?go=Support&pid=4440.

Removal efficiencies of modified samples in comparison of unmodified particle as per modification time (Fig. 15) and nature of modifier (Fig. 16) counter confirmed adsorption capacities of samples (Table 3). Maximum cobalt has been removed by OA4 (86.40 ± 1.3%) whereas minimum adsorption obtained from BA2 (50.04 ± 1.3%).

#### pH effect

**Figure 17:**
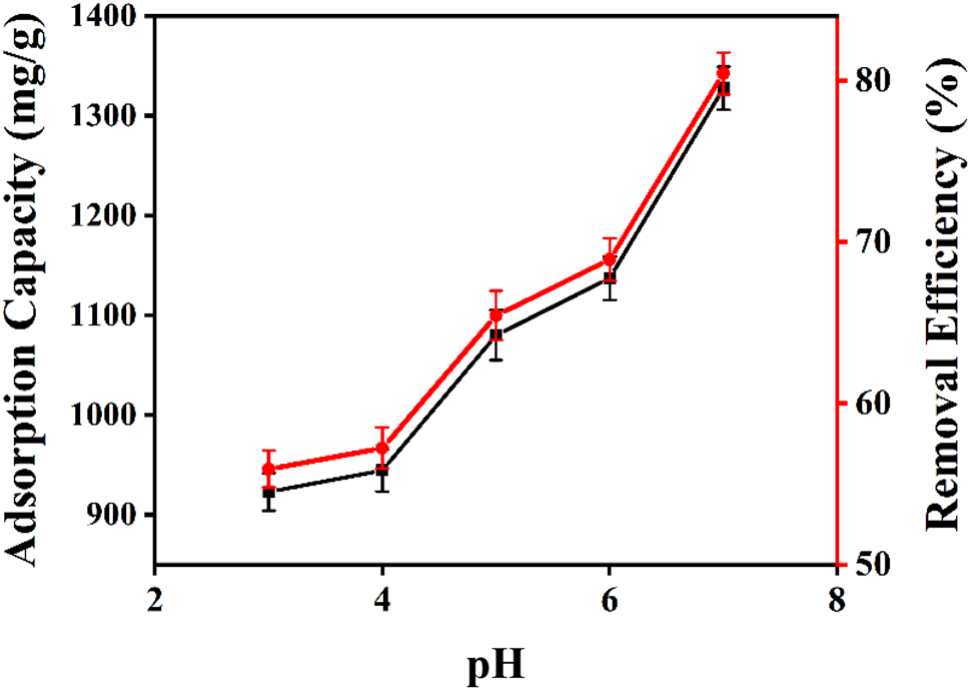
pH effect on Co adsorption capacity of OA4. OriginPro 2022 (64-bit) SR1 v9.9.0.225 https://www.originlab.com/index.aspx?go=Support&pid=4440.

OA4, as for its best output, has been tested out in the range of pH from 3 to 7, above this range precipitates of Co(II) ions formed as Co(OH)_2_^69^, and increase in adsorption with pH has been observed. Initial concentration (10 ml), adsorbent dosage (5 mg), stirring (200 rpm) and contact time(1 h) taken as predetermined conditions to tested out pH effect on adsorption behavior of prepared sample. It could be seen (Fig. 17, Table 4) that minimum adsorption obtained at pH 3 whereas maximum adsorption obtained at pH 7. Adsorption increasing trend (Fig. 17) seems to be supported as pH increases which is also in agreement of literature^53^.

**Table 4:** pH effect on OA4 adsorption capacity against Co(II) ions.

Sample	pH	Adsorption capacity(mg/g)	Removal efficiency (%)
OA4	3	923.01 ± 18.8	55.92 ± 1.1
	4	944.42 ± 21.1	57.22 ± 1.2
	5	1080.05 ± 25.1	65.44 ± 1.5
	6	1137.15 ± 21.8	68.90 ± 1.3
	7	1327.69 ± 21.6	80.44 ± 1.3

Other samples have also been tested in different pH environment, as pH increased from 4 to 6, some samples have shown an increment in adsorption capacity including AA2 (911.7 ± 26.4 mg/g), AA3 (1221.6 ± 23.1 mg/g), OA2 (1386.94 ± 21.4 mg/g), SA3 (1304.67 ± 21.5 mg/g).

## Conclusion

This research was aimed to prepare metal adsorbent from biowaste through facile chemical treatment, for effective an economical way of pollution control. Walnut shell powder has been treated with different organic acids at room temperature as well as at 550 °C. Thermal treatment of samples has shown maximum adsorption, same has been confirmed through CV analysis. The reason could be the development of magnetic behavior along with physical adsorption. OA4 sample has shown maximum adsorption (1371.4 ± 20.6 mg/g) and removal % (86.40 ± 1.3), signposting effective modification through OA. Analysis regarding effect of pH on adsorption capacity and % removal has shown incremental trend till pH 7. Results proved that biobased resources with suitable surface modification could be an economical, effective and efficient precursor for metal adsorbents in waste management applications.

## Data Availability

The datasets used and/or analyzed during the current study available from the corresponding author on reasonable request.
